# The Antimicrobial Peptide Human β-Defensin-3 Accelerates Wound Healing by Promoting Angiogenesis, Cell Migration, and Proliferation Through the FGFR/JAK2/STAT3 Signaling Pathway

**DOI:** 10.3389/fimmu.2021.712781

**Published:** 2021-09-14

**Authors:** Miho Takahashi, Yoshie Umehara, Hainan Yue, Juan Valentin Trujillo-Paez, Ge Peng, Hai Le Thanh Nguyen, Risa Ikutama, Ko Okumura, Hideoki Ogawa, Shigaku Ikeda, François Niyonsaba

**Affiliations:** ^1^Atopy (Allergy) Research Center, Juntendo University Graduate School of Medicine, Tokyo, Japan; ^2^Department of Dermatology and Allergology, Juntendo University Graduate School of Medicine, Tokyo, Japan; ^3^Faculty of International Liberal Arts, Juntendo University, Tokyo, Japan

**Keywords:** angiogenesis, human β-defensin, fibroblast, migration, proliferation, wound healing

## Abstract

In addition to its antimicrobial activity, the skin-derived antimicrobial peptide human β-defensin-3 (hBD-3) promotes keratinocyte proliferation and migration to initiate the wound healing process; however, its effects on fibroblasts, which are the major cell type responsible for wound healing, remain unclear. We investigated the role of hBD-3 in cell migration, proliferation and production of angiogenic growth factors in human fibroblasts and evaluated the *in vivo* effect of hBD-3 on promoting wound healing and angiogenesis. Following hBD-3 treatment, the mouse wounds healed faster and showed accumulation of neutrophils and macrophages in the early phase of wound healing and reduction of these phagocytes 4 days later. hBD-3-treated wounds also displayed an increased number of fibroblasts and newly formed vessels compared to those of the control mice. Furthermore, the expression of various angiogenic growth factors was increased in the hBD-3-treated wounds. Additionally, *in vitro* studies demonstrated that hBD-3 enhanced the secretion of angiogenic growth factors such as fibroblast growth factor, platelet-derived growth factor and vascular endothelial growth factor and induced the migration and proliferation of human fibroblasts. The hBD-3-mediated activation of fibroblasts involves the fibroblast growth factor receptor 1 (FGFR1)/Janus kinase 2 (JAK2)/signal transducer and activator of transcription 3 (STAT3) pathways, as evidenced by the inhibitory effects of pathway-specific inhibitors. We indeed confirmed that hBD-3 enhanced the phosphorylation of FGFR1, JAK2 and STAT3. Collectively, the current study provides novel evidence that hBD-3 might be a potential candidate for the treatment of wounds through its ability to promote wound healing, angiogenesis and fibroblast activation.

## Introduction

The skin is the most external organ that protects the body from physical and chemical environmental challenges. Therefore, once skin injury occurs, an immediate and coordinated response is initiated to repair the wound. The skin wound healing process consists of three distinct but overlapping stages, denoted as the inflammatory phase, proliferative phase, and remodeling phase, and involves several mediators, such as inflammatory cells, growth factors and cellular and extracellular elements (1). In the inflammatory phase, both neutrophils and macrophages are attracted to the wound site to ingest and kill microbes, remove damaged tissues and release proinflammatory cytokines and growth factors that recruit and activate fibroblasts and keratinocytes. In the proliferative phase, keratinocytes and fibroblasts proliferate and migrate to the wound site, where fibroblasts synthesize extracellular matrix (ECM) to form granulation tissues. In this phase, angiogenesis also plays an important role by supplying newly formed tissue. In the remodeling phase, ECM maturation and turnover and homeostasis are regulated by metalloproteinases (MMPs). In addition to the ECM, MMPs cleave many cytokines, growth factors and cytokine/growth factor receptors that regulate several steps of the wound healing process (2).

Angiogenesis is tightly regulated by a complex of angiogenic growth factors, such as epidermal growth factor (EGF), fibroblast growth factor (FGF), platelet-derived growth factors (PDGF), and vascular endothelial growth factor (VEGF). During the wound healing process, these angiogenic growth factors promote angiogenesis by binding to their respective receptors, such as EGF receptor (EGFR), FGF receptor (FGFR), PDGF receptor (PDGFR), and VEGF receptor (VEGFR). Following binding to their corresponding ligands, these receptors undergo phosphorylation, resulting in activation of the downstream signaling pathways, therefore leading to cell proliferation, migration, angiogenesis, and wound healing (3–6). Among the various downstream signaling pathways of angiogenic receptors, Janus kinase (JAK)/signal transducer and activator of transcription (STAT) pathway transmits extracellular signals to the nucleus for the transcription of numerous genes that promote cell proliferation, differentiation and angiogenesis. JAKs belong to the tyrosine kinase family of enzymes, and their activation leads to the transfer of extracellular signals provided by growth factors, cytokines, and chemokines (7). The JAK family comprises JAK1, JAK2, JAK3 and TYK2. Activation of STATs is typically mediated *via* the cytokine receptor associated with the JAK family of nonreceptor tyrosine kinases (8). To date, seven mammalian STAT family members, STAT1, STAT2, STAT3, STAT4, STAT5a, STAT5b and STAT6, have been identified (9, 10). In nonhealing chronic wounds, the levels of many angiogenic growth factors and their receptors are downregulated, therefore delaying wound healing (11–13). Consequently, there is an unmet need for the development of drugs capable of activating angiogenic growth factor receptors and their downstream cascades to promote wound healing.

Antimicrobial peptides not only kill invading pathogenic microorganisms but also contribute to host defense and homeostasis by exhibiting a wide range of immunomodulatory functions (14). Several types of antimicrobial peptides, including human β-defensins (hBDs), cathelicidin LL-37, psoriasin (S100A7), and ribonuclease RNase-7, have been identified in human skin. Some are permanently expressed in healthy skin, and some are increased following infections, injury, proinflammatory cytokines and ultraviolet B irradiation (14). The hBD family is one of the most important antimicrobial peptide families found in human skin, and these peptides are well known for their wide range of microbicidal activities and immunomodulatory properties. To date, six members of hBDs, namely, hBD-1 to hBD-6, have been identified in the human body. Among them, hBD-1 to hBD-4 are primarily found in the epithelia of the skin, eyes, and oral, respiratory and urogenital tracts, while hBD-5 and hBD-6 are only present in the epididymis (15, 16). Among hBDs present in the skin, only hBD-1 is constitutively expressed, whereas the expression of hBD-2 to hBD-4 is enhanced upon infection or inflammation (17). In addition to killing a broad spectrum of invading microorganisms such as bacteria, viruses and fungi, hBDs also act as immunoregulators and represent a link between innate and adaptive immune responses. Various hBD-mediated immunomodulatory functions include the regulation of pro- and anti-inflammatory responses, neutralization of lipopolysaccharide, chemoattraction and activation of numerous immune cells, induction of cell proliferation, promotion of wound healing and maintenance of the skin barrier (17). It has been documented that hBDs activate various cell types, such as mast cells, neutrophils, macrophages and keratinocytes, which largely contribute to wound healing (14). Furthermore, hBD-2 and hBD-3 expression levels are upregulated by keratinocytes at skin wound sites and promote cell migration and proliferation (14, 18). hBD-induced keratinocyte proliferation and migration are mediated through phosphorylation of the EGFR and STAT proteins (19). Notably, the accelerating effects of hBDs on wound healing are not restricted to the skin but extend to other tissues, such as the eye and gut (20–22). To date, the effects of hBDs on fibroblasts, which play a crucial role in wound healing, have not been reported.

In the present study, among the hBDs tested, only hBD-3 showed the strongest stimulatory effect on human fibroblasts. hBD-3 induced the production of various angiogenic growth factors, such as FGF, PDGF and VEGF, promoted fibroblast migration and proliferation and accelerated *in vitro* wound healing. hBD-3-mediated activation of fibroblasts involved the FGFR1/JAK2/STAT3 signaling pathway. Moreover, hBD-3 markedly accelerated *in vivo* wound healing, increased the number of neutrophils, macrophages and fibroblasts at the wound site, and enhanced the formation of vessels and the expression of angiogenic growth factors in mice. These findings provide novel evidence that hBD-3 may contribute to wound healing *via* activation of fibroblasts.

## Materials and Methods

### Reagents

Antimicrobial peptide hBD-3 was purchased from Peptide Institute (Osaka, Japan), whereas recombinant mouse β-defensin 14 (mBD-14) was obtained from ProSpec (Rehovot, Israel). Fruquintinib, imatinib mesylate and mitomycin C were obtained from Sigma-Aldrich (St Louis, MO). Cryptotanshinone and AZD1480 were purchased from Selleck Chemicals (Houston, TX). SSR 128129E was purchased from Cayman Chemical (Ann Arbor, MI), recombinant basic FGF and VEGF were purchased from Peprotech (Cranbury, NJ). Antibodies against phosphorylated and unphosphorylated FGFR1, JAK1, JAK2, JAK3, STAT1 and STAT3 and RIPA buffer that consisted of 20 mM Tris-HCl (pH 7.5), 150 mM NaCl, 1 mM Na_2_ EDTA, 1 mM EGTA, 1% NP-40, 1% sodium deoxycholate, 2.5 mM sodium pyrophosphate, 1 mM β-glycerophosphate, 1 mM Na_3_VO_4_ and 1 µg/ml leupeptin were obtained from Cell Signaling Technology (Beverly, MA). Type 1 collagen was purchased from Calbiochem (La Jolla, CA). Rabbit polyclonal antibody against ionized calcium-binding adapter molecule 1 (Iba1) was purchased from Fujifilm Wako Pure Chemical Corporation (Tokyo, Japan). Rabbit polyclonal antibody against myeloperoxidase (MPO) was obtained from CiteAb (Bath, United Kingdom). Mouse monoclonal antibody against heat shock protein 47 (HSP47) was obtained from Santa Cruz Biotechnology (Dallas, TX), whereas rabbit polyclonal antibody against mBD-14 was purchased from MyBioSource (San Diego, CA). Rabbit polyclonal antibody against CD163 was obtained from Proteintech (Rosemont, IL). Rabbit polyclonal antibody against CD80 was obtained from Abcam (Cambridge, UK).

### Animal Experiments

Eight-week-old male C57BL/6 mice (Japan SLC, Inc., Ibaraki, Japan) were maintained under specific pathogen-free conditions and fed a standard diet and water *ad libitum*. All procedures were approved and performed in accordance with the Institutional Animal Care and Use Committee of Juntendo University School of Medicine (approval number: 2021255) and complied with the National Institutes of Health Guide for the Care and Use of Laboratory Animals. Following anesthesia with 2.5% isoflurane, hairs of the dorsal skin were shaved, and two 6-mm-diameter full-thickness wounds were created in the dorsal skin of mice using a circular biopsy punch under aseptic conditions. Ring-shaped silicone splints were sutured to the wound perimeter to avoid and prevent contraction. The mice were randomized into a control group that was treated with 0.01% acetic acid (solvent) and an hBD-3 group. In total, 200 µg/ml hBD-3 or mBD-14 in 20 µl of 0.01% acetic acid was topically applied every 2 days until the wounds were completely healed. After surgery, the wounds were covered with a hydrocolloid dressing (Tegaderm; 3 M Health Care, Tokyo, Japan) and cleaned with normal saline before each treatment. Digital photographs of each wound were taken, and the wound areas were calculated using ImageJ software (NIH, Bethesda, MD). After anesthesia, the mice were sacrificed, and wound skin samples were collected for further experiments.

### Immunohistochemistry

Wound skin tissues were obtained after fixation with 10% paraformaldehyde (pH 7.4) in phosphate-buffered saline (PBS), processed and embedded in paraffin. The sections were deparaffinized, hydrated and blocked with normal goat serum (1:20 dilution) for 30 minutes at room temperature before being incubated at 4°C overnight with anti-MPO antibody (1:1000 dilution) for neutrophil identification, anti-Iba1 antibody (1:2000 dilution) for macrophage detection, anti-HSP47 antibody (1:1000 dilution) for fibroblast identification, anti-mBD-14 antibody (1:500 dilution), anti-CD80 antibody (1:100 dilution) and anti-CD163 (1:100 dilution) for M1 and M2 identification, respectively. After three washes in PBS, the sections were allowed to react with horseradish peroxidase-conjugated streptavidin (1:300 dilution) for 30 minutes at room temperature. Diaminobenzidine tetrahydrochloride solution was then added and incubated at room temperature for 1 minute (for anti-Iba-1 antibody, anti-CD163 antibody), 2 minutes (for anti-MPO antibody and for anti-mBD-14 antibody), and 3 minutes (for anti-HSP47 antibody) and 5 minutes (for anti-CD80 antibody). Sections were stained with hematoxylin, dehydrated in an ethanol gradient, cleared in dimethyl benzene, and mounted. Images were acquired using a phase-contrast microscope (Keyence, Osaka, Japan) at 400× magnification, and images were recorded. The wound tissue areas were measured using ImageJ software. The number of MPO-, Iba1-, HSP47-, CD80- and CD163-positive cells was counted in three microscopic fields for each sample in the fields with the highest numbers of target cell.

### Cell Culture

Normal human dermal fibroblasts were purchased from Lifeline Cell Technology (Osaka, Japan) and cultured in FibroLife serum-free medium (Lifeline Cell Technology) containing L-glutamine (7.5 mM), human basic FGF (5 ng/ml), insulin (5 μg/ml), ascorbic acid (50 μg/ml), hydrocortisone (1 μg/ml), gentamycin (30 μg/ml), amphotericin B (15 ng/ml) and fetal bovine serum (2% vol/vol). All experiments were performed using subconfluent cells (from 60% to 80% confluence) grown in FibroLife serum-free medium without supplements but with antibiotics.

### RNA Extraction and Real-Time PCR

Wound skin tissues were immediately stored in RNAlater solution (Ambion, Austin, TX) to stabilize and protect the RNA integrity before disruption in QIAzol Lysis Reagent (Qiagen, Hilden, Germany). Total RNA from wound tissues was extracted using the RNeasy Plus Universal Mini kit (Qiagen, Hilden, Germany), while RNA extraction from fibroblasts was performed using an RNeasy Plus Micro kit (Qiagen) according to the manufacturer’s instructions. cDNA was synthesized from 1 μg of total RNA using the ReverTra Ace qPCR RT kit (Toyobo, Osaka, Japan). Real-time PCR was performed using a QuantiNova SYBR Green PCR kit (Qiagen), and cDNA was amplified and detected using the StepOne Plus Real-time PCR System (Applied Biosystems). The primer sequences used for *Pdgfc* were forward: 5′-GTGGAGGAAATTGTGCCTGT-3′ and reverse: 5′-TCCAGAGCCACATCAGTGAG-3′, for *Fgf2* were forward: 5′-GGACGGCTGCTGGCTTCTAA-3′ and reverse: 5′-CCAGTTCGTTTCAGTGCCACATAC-3′, for *Vegfa* were forward: 5′-GTGCACTGGACCCTGGCTTTA-3′ and reverse: 5′-GGTCTCAATCGGACGGCAGTA-3′, for *Rps18* were forward: 5′-TTCTGGCCAACGGTCTAGACAAC-3′ and reverse: 5′-CCAGTGGTCTTGGTGTGCTGA-3′, and for *Defb14* were forward: 5′-ATCTTGTTCTTGGTGCCTGC-3′, and reverse: 5′-CTTCTTTCGGCAGCATTTTC-3′. All primers were obtained from Thermo Fisher Scientific (Waltham, MA). The target RNA level was normalized to the endogenous *Rps18* reference, and the changes in gene expression were reported as fold increases relative to vehicle.

### Enzyme-Linked Immunosorbent Assay

Subconfluent fibroblasts were stimulated with 10-20 µg/ml hBD-3 for 3-48 hours, and the cell-free supernatants from cultures of stimulated cells or nonstimulated control cells were collected and used for the quantification of FGF, PDGF and VEGF by appropriate ELISA kits obtained from R&D Systems (Minneapolis, MN). ELISAs were performed following the recommendations of the supplier. In some experiments, cells were pretreated with cryptotanshinone, AZD1480 or SSR 1281289E for 2 hours before stimulation with hBD-3, and ELISA was performed as described above.

### Chemotaxis Assay

Fibroblast migration was assayed using a modified Boyden chamber consisting of a 48-well microchamber (Neuro Probe, Gaithersburg, MD). Subconfluent fibroblasts were trypsinized, and a volume of 50 µl containing 5.0 × 10^4^ cells was loaded into the upper chambers, which were separated from the lower chambers by a polyvinylpyrrolidone-free polycarbonate membrane with an 8-µm pore size (Neuro Probe) and precoated with 10 µg/ml type I collagen at 37°C for 2 hours. The lower chambers contained 27 µl of various doses of hBD-3 or vehicle. After a 6-hour incubation at 37°C in a CO_2_ incubator, the membrane was removed from the chamber, and the cell side was rinsed in PBS drawn across with a wiper blade to remove the nonmigrated cells. The membrane was then stained with Diff-Quick (Kokusai Shiyaku, Kobe, Japan) according to the manufacturer’s instructions. Cells that had migrated and adhered to the lower side of the membrane were counted in 3 randomly selected high-power fields under a laser scanning microscope 700 (Zeiss, Jena, Germany). In some experiments, cells were pretreated with various inhibitors for 2 hours before being added to the upper chambers, and the chemotaxis assay was performed as described above.

### *In Vitro* Scratching Assay

Fibroblasts were seeded in a collagen I-coated 96-well plate at a density of 5×10^4^ cells/well for 6 hours at 37°C. Confluent fibroblast monolayers were then wounded using a wound maker (Essen BioScience, Ann Arbor, MI) to create a uniform cell-free zone in each well and washed with PBS to remove the detached cells. In all experiments, fibroblasts were pretreated with 10 µg/ml mitomycin C for 2 hours before stimulation with hBD-3 for 24-48 hours to exclude the influence of hBD-3-mediated cell proliferation on migration. After stimulation, fibroblasts were stained with 0.5% crystal violet (Fujifilm, Tokyo, Japan), and images were recorded using a phase-contrast microscope (Keyence). Wound area was measured using ImageJ software. In some experiments, cells were pretreated with inhibitors for 2 hours before stimulation with hBD-3.

### Cell Proliferation Assay

Cell proliferation was assessed by Cell Counting Kit-8 (CCK-8) purchased from Dojindo Laboratories (Kumamoto, Japan) following instructions from the manufacturer. Fibroblasts were seeded into 96-well plates at a density of 1.0×10^4^ cells/well and cultured for 24 hours before stimulation with hBD-3 for 72 hours. After stimulation, 10 µl of CCK-8 solution was added to each well, and the cells were incubated for 1 hour at 37°C. The amount of formazan dye was measured by absorbance at 450 nm using a microplate reader. In some experiments, cells were pretreated with various inhibitors for 2 hours before stimulation with hBD-3.

### Western Blot

Following stimulation, fibroblasts were lysed with RIPA buffer, and the proteins were extracted from the cells and quantified using Precision Red Protein Assay Reagent (Cytoskeleton, Denver, CO). Equal amounts of protein were separated in an 8% to 12% SDS-PAGE gel followed by transfer to methanol-preactivated polyvinylidene difluoride membranes (Millipore, Billerica, MA) for 50 minutes. The membranes were then blocked in Immuno-Block buffer (KAC, Hyogo, Japan.) for 1 hour at room temperature and incubated with primary antibodies against MMP-2 (1:1000 dilution), MMP-9 (1:2000 dilution), phosphorylated FGFR1 (1:500 dilution), STAT1 (1:500 dilution), STAT3 (1:1000 dilution), JAK1 (1:500 dilution), JAK2 (1:500 dilution), JAK3 (1:500 dilution), unphosphorylated FGFR1, STAT1, STAT3, JAK1, JAK2 and JAK3 (each 1:1000 dilution) overnight at 4°C, according to the manufacturer’s instructions. After washing, the membranes were developed with Luminata Forte Western HRP substrate (Millipore, Billerica, MA) for detection and visualized using Fujifilm LAS-4000 Plus (Fujifilm). The intensity of bands was quantified using the software program ImageJ Gauge (LAS-1000plus, Fujifilm) to allow correction for protein loading.

### Statistical Analysis

Statistical analysis was performed by one-way or two-way analysis of variance (ANOVA) with multiple comparisons test or Student’s *t*-test using GraphPad Prism version 6.0 (San Diego, CA). Values are presented as the mean ± standard deviation (SD). *P* < 0.05 was considered significant.

## Results

### hBD-3 Accelerates Wound Healing *In Vivo*

**Figure 1:**
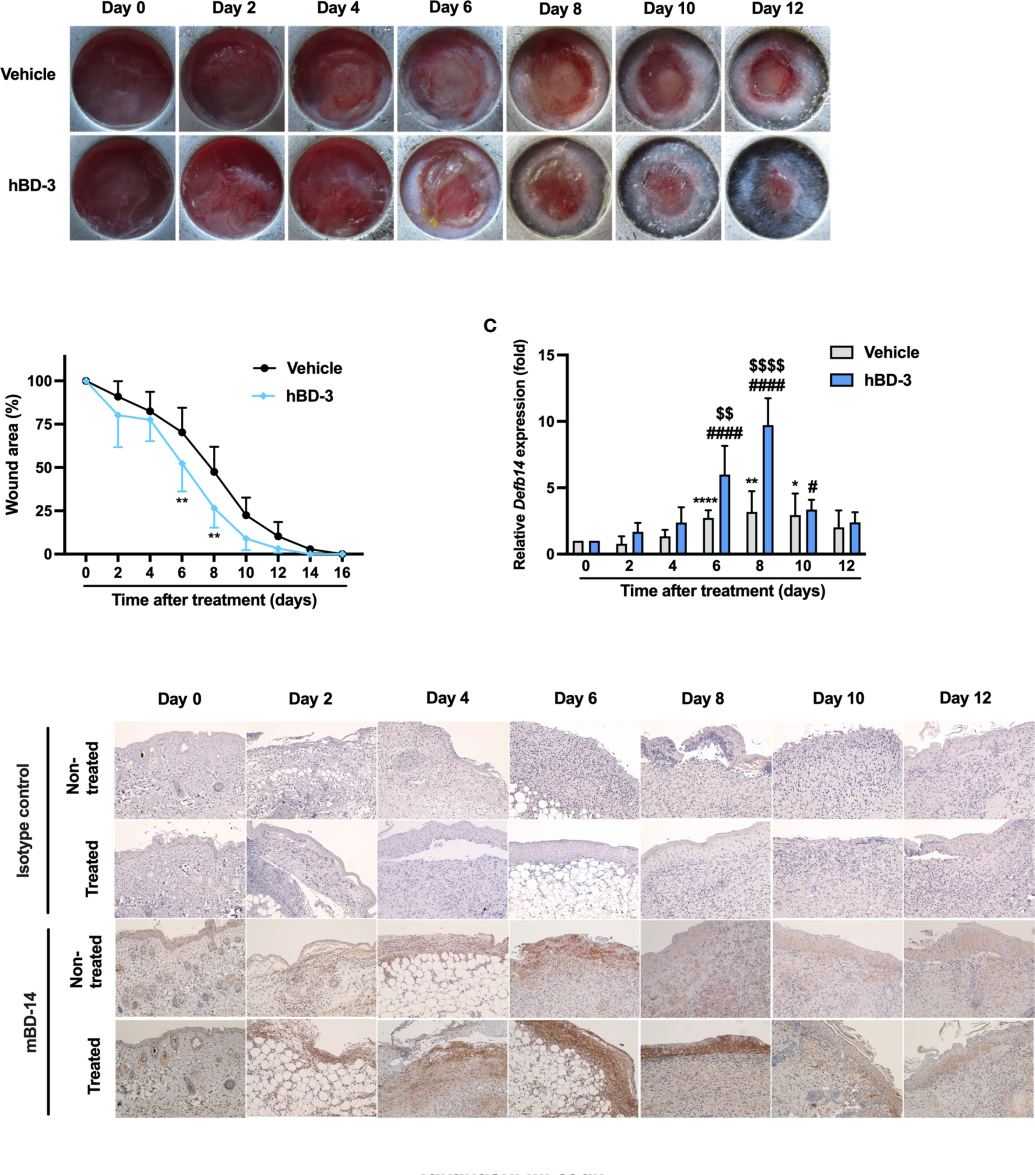
hBD-3 accelerates wound closure *in vivo.* Dorsal full-thickness skin wounds were created on mice and then topically treated with 0.01% acetic acid (vehicle) and 200 µg/ml hBD-3. **(A)** Representative images of skin wounds from day 0 to day 12. **(B)** The average wound area was calculated using ImageJ software. The *P* value was evaluated using one-way ANOVA with Tukey’s multiple comparisons test. *P < 0.05 and **P < 0.01 between the vehicle-treated wounds and the hBD-3-treated wounds. *n* = 3 wound areas/group. **(C)** mRNA was extracted from the wound tissues and the mRNA expression of Defb14 (mBD-14) was detected by quantitative real-time PCR analysis using SYBR Premix Ex Taq. The *P* value was determined by one-way ANOVA with Tukey’s multiple comparisons test. *P < 0.05, **P < 0.01 and ****P < 0.0001 for comparisons between the vehicle-treated wounds. ^#^ *P* < 0.05 and ^####^ *P* < 0.0001 for comparisons between hBD-3-treated wounds. ^$$^ *P* < 0.01 and ^$$$$^ *P* < 0.0001 for comparisons between the vehicle-treated and hBD-3-treated wounds. *n* = 3. **(D)** Representative images of mBD-14 staining and isotype control staining in treated and nontreated wounds from day 0 to 12. Original magnification: 20×.

To investigate the effect of hBD-3 on skin wound healing, we first used an excisional wound model to observe the healing process for 16 consecutive days in C57BL/6 mice. We used a wound splinting model to prevent local contraction because with this model, the wound heals through granulation and re-epithelization, similar to the wound healing process in humans. In the preliminary dose-dependent experiments using 40−400 µg/ml hBD-3, we observed that 200 µg/ml was the most effective concentration for accelerating wound healing. Therefore, this concentration was used in further *in vivo* experiments. As shown in **Figure 1A**, the wounds treated with hBD-3 displayed considerable signs of wound healing and healed faster than the vehicle-treated wounds. The hBD-3-treated wounds started to heal on day 6 and completely healed on day 12 post-injury. **Figure 1B** shows the percentage of wound area. The wound percentage was significantly lower in the hBD-3-treated groups than in the vehicle groups on days 6, 8 and 10 post-injury, suggesting that hBD-3 accelerated wound healing *in vivo*. We confirmed that mBD-14, a mouse ortholog of hBD-3 (23), also accelerated wound healing in mice with almost the same potency as seen for hBD-3 (**Figure S1**).

A previous study reported that skin injury induces hBD-3 expression (24). Therefore, we evaluated the basal expression of mBD-14/hBD-3 at both mRNA and protein levels. As seen in **Figure 1C**, the basal mRNA expression of mBD-14 was markedly increased from day 6 to day 10 in nontreated groups compared to day 0, and this expression was further enhanced in hBD-3-treated wounds. Meanwhile, immunohistochemical analysis showed that nontreated wounds had the highest basal expression of mBD-14 on day 4 and day 6, and the treatment of wounds with hBD-3 further increased this expression from day 2 to day 10, with wounds on day 6 and day 8 showing the stronger staining of mBD-14 compared to vehicle groups (**Figure 1D**).

### hBD-3 Promotes Angiogenesis and Increases the Production of Angiogenic Growth Factors

**Figure 2:**
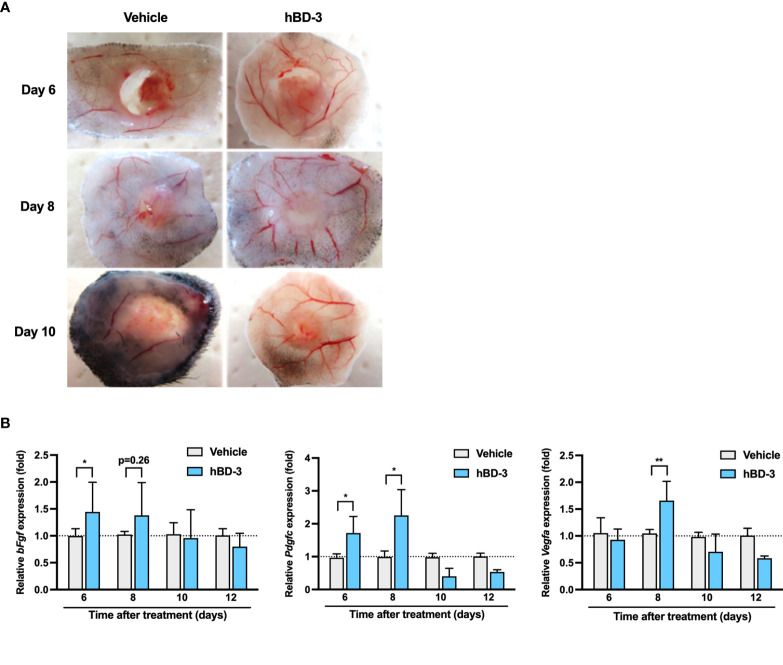
hBD-3 promotes angiogenesis and induces the production of angiogenic growth factors. **(A)** Representative images of the macroscopic appearance of newly formed blood vessels at the wound sites on days 6, 8 and 10 post-injury in the mice treated with 0.01% acetic acid (vehicle) and 200 µg/ml hBD-3. **(B)** Biopsies were obtained from the dorsal skin wound tissues, and the mRNA expression of *bFgf, Pdgf*, and *Vegfa* was evaluated by quantitative real-time PCR analysis using SYBR Premix Ex Taq. The *P* value was calculated using one-way ANOVA with Tukey’s multiple comparisons test. *P < 0.05 and **P < 0.01 for comparisons between the vehicle-treated and hBD-3-treated wounds. *n* = 3.

Angiogenesis is one of the critical processes for wound healing. Given that hBD-3 accelerated wound healing *in vivo*, we hypothesized that this peptide might also contribute to the promotion of angiogenesis during wound healing. The analysis of subcutaneous wound tissues collected on days 6, 8 and 10 post-injury revealed that the wounds treated with hBD-3 had remarkably increased new vessel formation. Compared to the vehicle-treated groups, the hBD-3-treated groups displayed increased vessel size and number of vessels in subcutaneous tissues (**Figure 2A**).

Given that angiogenesis is tightly regulated by various angiogenic growth factors, the effect of hBD-3 on the expression of angiogenic growth factors was investigated. As observed in **Figure 2B**, hBD-3-treated wound tissues showed significantly higher mRNA expression of FGF on day 6, PDGF on days 6 and 8 and VEGF on day 8 post-injury than that of the control tissues. hBD-3 treatment did not have any effect on other growth factors, such as epidermal growth factor and transforming growth factor (data not shown).

### hBD-3 Initiates the Inflammatory Phase of Wound Healing and Decreases Inflammatory Responses

**Figure 3:**
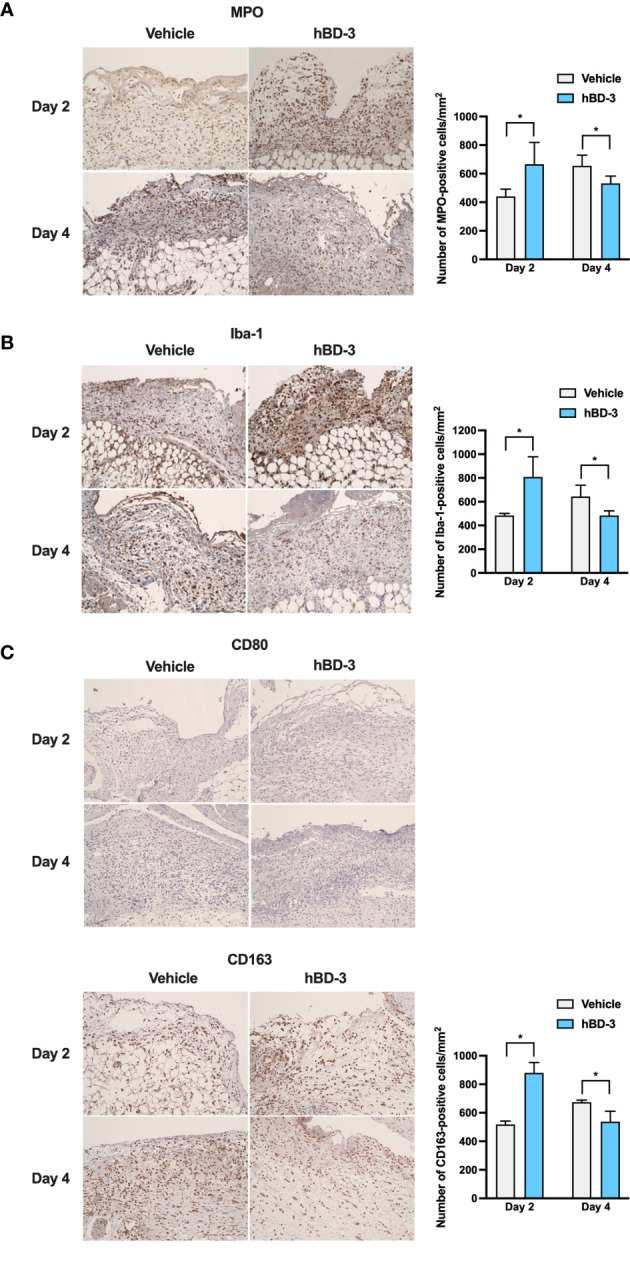
hBD-3 accelerates the inflammatory phase of wound healing and decreases inflammatory responses. Representative images of skin wound biopsies from mice treated with 0.01% acetic acid (vehicle) and 200 µg/ml hBD-3. On day 2 and day 4 post-injury, sections were immunohistochemically stained with **(A)** anti-myeloperoxidase (MPO) antibody for neutrophil detection, **(B)** with anti-Iba-1 antibody for macrophage detection, and **(C)** with anti-CD80 (upper panels) and anti-CD163 (lower panels) antibodies for M1 and M2 macrophage detection followed by counterstaining with hematoxylin. MPO-, Iba-1- and CD163-positive cells were detected in both the epidermis and dermis of the wounds. Original magnification: 20×. Number of MPO-, Iba-1- and CD163-positive cells were counted and shown in right panels. The *P* value was calculated using Student’s *t* test. *P < 0.05 for comparisons between the vehicle-treated and hBD-3-treated wounds. *n* = 3.

In the inflammatory phase, inflammatory cells such as neutrophils and macrophages are recruited to the wound site to cleanse the wound (25). In this study, we chose MPO and Iba1 as markers of neutrophils and macrophages, respectively. Iba1 is recognized as a panmacrophage marker and has been widely used to identify macrophages within skin (26, 27). As observed in **Figure 3A**, immunohistochemistry showed that on day 2 post-injury, a higher number of neutrophils was observed in the wound areas treated with hBD-3 than in those of the control groups treated with vehicle alone. Interestingly, on day 4 after injury, the number of neutrophils was significantly reduced in the hBD-3-treated groups compared with the control groups. Quantification is shown in right panel. Similarly, as observed in **Figure 3B** (quantification shown in right panel), the number of macrophages at the wound site treated with hBD-3 was higher on day 2 post-injury, and this number decreased on day 4 compared to that of the vehicle-treated wounds. Macrophages have been divided into M1 and M2 subtypes. We used CD80 as a marker of pro-inflammatory M1 phenotype (28), and CD163 for identification of anti-inflammatory M2 phenotype (29). While M1 positive macrophages were not detected neither at nontreated nor treated wound areas (**Figure 3C**, upper panels), M2 positive cells noticeably increased at the wound sites treated with hBD-3 on day 2 and this number declined on day 4 (**Figure 3C**, lower panels, quantification shown in right panel). Successful wound healing requires the shifting of macrophages from pro-inflammatory M1 phenotype to anti-inflammatory M2 phenotype with wound healing capacity (30, 31). Overall, this observation suggests that hBD-3 initiates the inflammatory phase of the wound healing process by inducing the infiltration of neutrophils and macrophages and decreases inflammation by later eliminating these cells during wound healing.

### hBD-3 Promotes Fibroblast Accumulation *In Vivo* and Migration and Proliferation *In Vitro*

**Figure 4:**
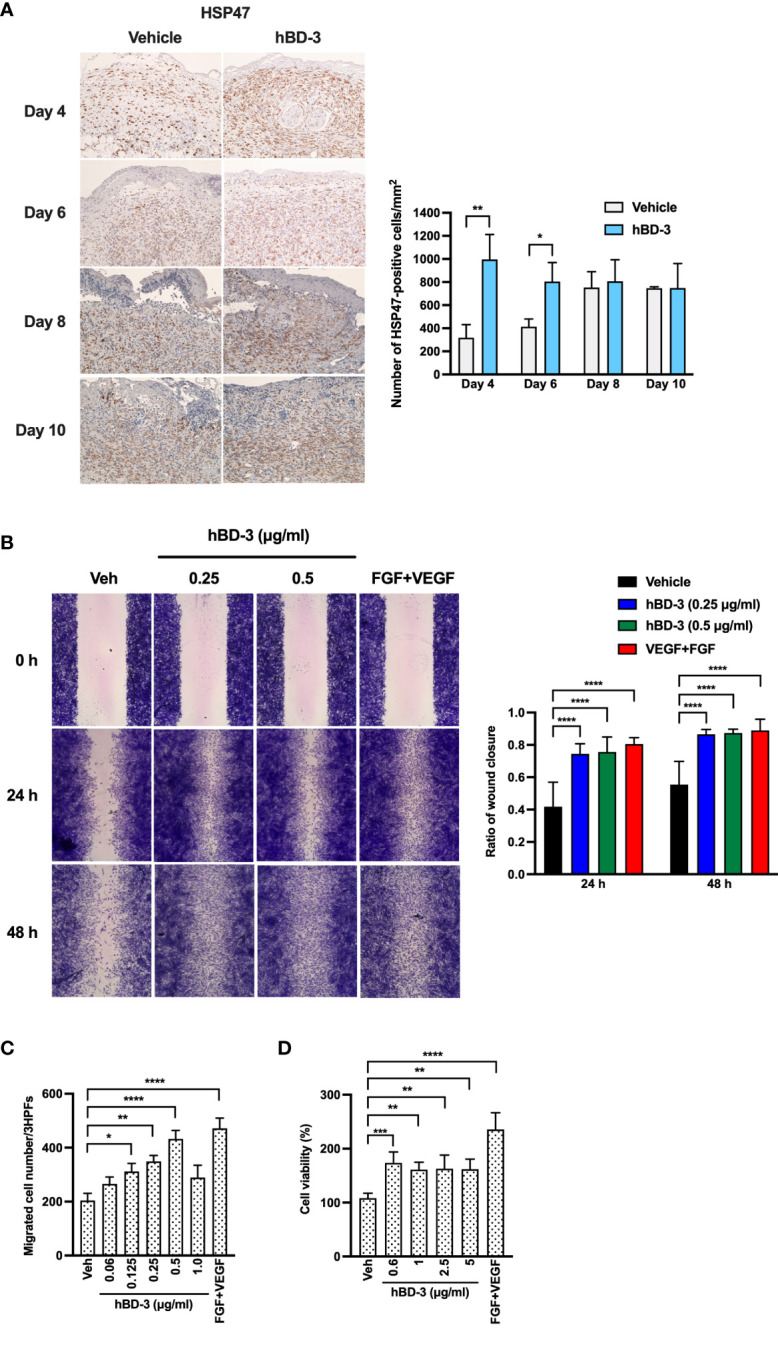
hBD-3 enhances fibroblast accumulation *in vivo* and migration and proliferation *in vitro*. **(A)** Representative images of skin wound biopsies from mice treated with 0.01% acetic acid (vehicle) and 200 µg/ml hBD-3. On day 4, 6, 8 and 10 post-injury, sections were immunohistochemically stained with anti-heat shock protein 47 (HSP47) antibody for fibroblast detection and then counterstained with hematoxylin. HSP47-positive cells were detected in both the epidermis and dermis of the wounds. Original magnification: 20×. Number of HSP47-positive cells were counted and shown in right panel. The *P* value was calculated using Student’s *t* test. *P < 0.05 and **P < 0.01 for comparisons between the vehicle-treated and hBD-3-treated wounds. *n* = 3. **(B)** Human primary fibroblasts were treated with 10 µg/ml mitomycin C for 2 hours, and a scratch assay was performed. Following treatment with 0.01% acetic acid (vehicle) and 0.25 and 0.5 µg/ml hBD-3 and FGF+VEGF (100 ng/ml each) for 24 and 48 hours, fibroblasts were stained with crystal violet, and images were recorded. Left panels are representative images of the scratch assay, and right panels show average data calculated using ImageJ software. **(C)** Various doses of hBD-3 were added to the lower chambers, and fibroblasts were added in the upper chambers of the chemotaxis microchamber. Following a 6-hour incubation, the membrane was stained, and migrated cells attached to the bottom surface were fixed and then stained with Diff-Quick. Migrated cells were counted in 3 random high-power fields (HPFs) under a light microscope. **(D)** Cells were stimulated with the indicated doses of hBD-3 or FGF+VEGF (100 ng/ml each) for 72 hours. Cell proliferation was assessed using a CCK-8 kit. The *P* value was calculated using one-way ANOVA with Tukey’s multiple comparisons test. *P < 0.05, **P < 0.01, ***P < 0.001, and ****P < 0.0001 for comparison with vehicle and hBD-3. *n* = 3.

The second phase of the wound healing process consists of the proliferative phase, which mainly involves fibroblast migration and proliferation (6). HSP47, rather than α-smooth muscle actin (α-SMA), was used as a marker of fibroblasts because we aimed to identify all fibroblasts instead of only myofibroblasts that express α-SMA (32, 33). Notably, the α-SMA antibody may be less specific, as it also stains vascular smooth muscle cells in granulation tissue (34). Immunohistochemistry showed that on day 4 and day 6 post-injury, the wounds treated with hBD-3 displayed a higher number of fibroblasts than wounds treated with vehicle alone. Although fibroblast number was also increased on day 8 and day 10 post-injury, there was not statistically significant difference between treated and nontreated groups (**Figure 4A**, quantification shown in right panel). This finding suggests that hBD-3 might promote fibroblast proliferation and/or migration at the wound site. Therefore, we first used a scratch assay to assess the ability of hBD-3 to induce fibroblast migration *in vitro* using primary human dermal fibroblasts in the presence of mitomycin C to exclude the influence of proliferation on wound healing. Compared to the vehicle-treated cells, the cells stimulated with hBD-3 for 24 and 48 hours rapidly migrated and covered the wound area. This effect was comparable to that induced by FGF+VEGF, which was used as a positive control (**Figure 4B**). Quantification of wound closure is shown in the right panel and illustrates significantly accelerated wound closure following treatment of cells with 0.25 and 0.5 µg/ml hBD-3. We observed that high doses of hBD-3 did not further increase wound closure (data not shown).

The ability of hBD-3 to mediate cell migration was further confirmed by an *in vitro* chemotaxis assay. We found that hBD-3 markedly caused cell migration in a bell-shaped dose-response manner, with 0.5 µg/ml being the optimal concentration. Higher hBD-3 concentrations reduced fibroblast migration (**Figure 4C**). The observation that hBD-3 accelerated wound healing in the presence of mitomycin C indicates that hBD-3-promoted wound healing might be mainly attributed to cell migration. To further determine how hBD-3 promotes wound healing, we investigated the effect of hBD-3 on cell proliferation using a CCK-8 assay kit. As shown in **Figure 4D**, noticeably increased proliferation of fibroblasts was observed when cells were treated with hBD-3. An approximately twofold increase in proliferation was found in fibroblasts stimulated with hBD-3, while the positive control of FGF+VEGF resulted in a threefold increase. Thus, hBD-3 induced both cell migration and proliferation, which are indispensable steps for the promotion of wound healing.

### hBD-3 Induces the Production of Angiogenic Growth Factors and the Expression of MMP-2 by Human Fibroblasts

**Figure 5:**
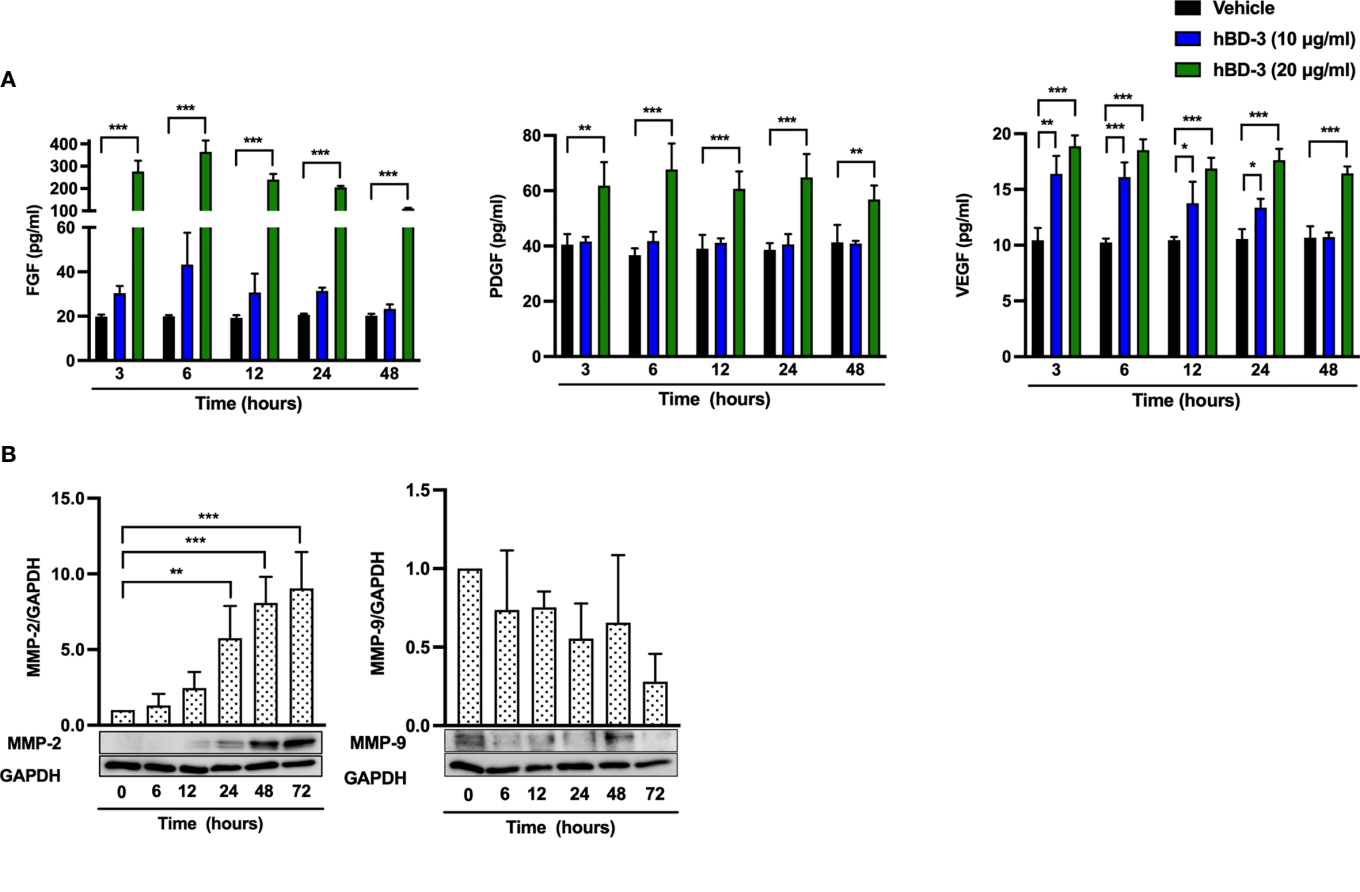
hBD-3 increases the production of angiogenic growth factors and the expression of MMP-2 in fibroblasts. **(A)** Fibroblasts were stimulated with 10-20 µg/ml hBD-3 or 0.01% acetic acid (vehicle) for 48 hours, and the amounts of VEGF (left panel), PDGF (middle panel) and FGF (right panel) released in the culture supernatants were determined by ELISAs. **(B)** Fibroblasts were stimulated with 20 μg/ml hBD-3 for 6 to 72 hours and subjected to Western blotting using antibodies against MMP-2 and MMP-9. Bands were quantified using densitometry. The *P* value was calculated using one-way ANOVA with Tukey’s multiple comparisons test. *P < 0.05, **P < 0.01, and ***P < 0.001 for comparisons between vehicle and hBD-3. *n* = 3.

Because hBD-3 promoted angiogenesis and elevated the expression of angiogenic growth factors *in vivo* and given that the fibroblast number was increased at the wound site, we examined the effect of hBD-3 on the production of angiogenic growth factors in human fibroblasts. Following stimulation with hBD-3 for 3 to 48 hours, the amounts of FGF, PDGF and VEGF in the cell supernatants were evaluated by ELISAs. Both 10 µg/ml and 20 µg/ml hBD-3 enhanced the production of VEGF in a dose-dependent fashion, while only 20 µg/ml hBD-3 markedly increased the production of FGF and PDGF at all time points examined (**Figure 5A**).

MMP-2 and MMP-9 are enzymes that play an important role in regulating angiogenesis during wound healing (35, 36). MMP-2 is essential for angiogenesis and prolonged matrix remodeling, while MMP-9 is involved in granulation tissue remodeling (37). Stimulation of human fibroblasts with hBD-3 resulted in significantly increased expression of MMP-2, which was detected from 24 hours and lasted until 72 hours. In contrast, MMP-9 expression was not changed by hBD-3 treatment (**Figure 5B**), suggesting that MMP-2 but not MMP-9 might be involved in hBD-3-mediated angiogenesis and wound healing.

### Activation of FGFR Is Required for the hBD-3-Mediated Production of Angiogenic Growth Factors, Migration, and Proliferation in Fibroblasts

**Figure 6:**
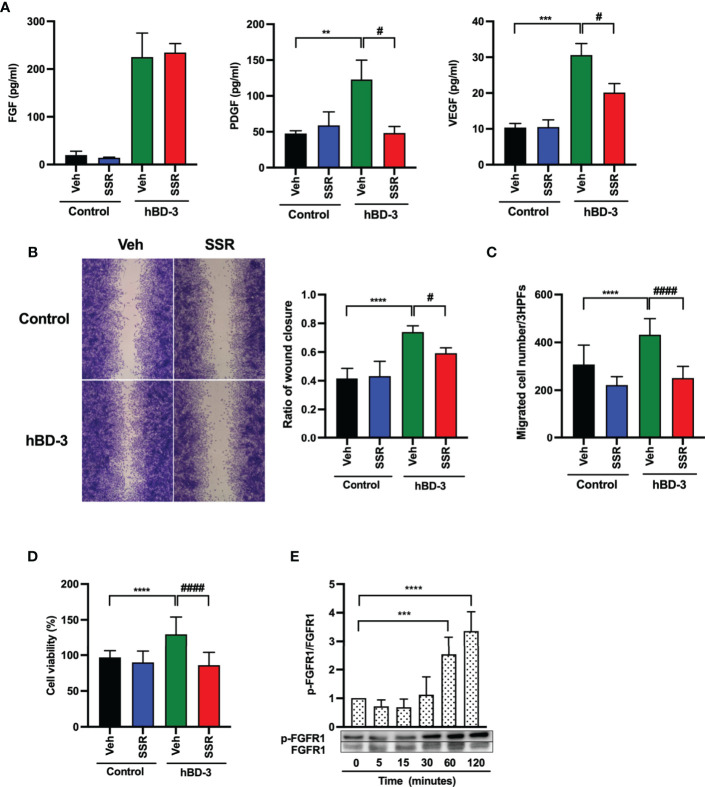
Activation of FGFR1 is required for hBD-3-mediated production of angiogenic growth factors, migration and proliferation of fibroblasts. Human dermal fibroblasts were pretreated with 0.1% DMSO (vehicle) or 1 µM SSR 128129E (SSR) for 2 hours and then exposed to 0.01% acetic acid (control) or hBD-3. **(A)** The levels of VEGF, PDGF and FGF in the culture supernatants from pretreated cells stimulated for 48 hours with 20 µg/ml hBD-3 were measured by appropriate ELISAs. **(B)** An *in vitro* wound scratch assay in pretreated fibroblasts stimulated with solvent (control) or 0.25 µg/ml hBD-3 for 48 hours was performed. Left panels show representative images, while the right panel represents the average of wound areas analyzed using ImageJ software. **(C)** Pretreated fibroblasts were also added to the upper wells of the microchamber and allowed to migrate for 6 hours towards solvent (control) or 0.5 µg/ml hBD-3. Migrated cells were counted in 3 random high-power fields (HPFs) under a light microscope. **(D)** Pretreated fibroblasts were stimulated with 1 µg/ml hBD-3 for 72 hours, and cell proliferation was assessed using the CCK-8 kit. **(E)** Fibroblasts were stimulated with 20 µg/ml hBD-3 for 5 minutes to 120 minutes and subjected to Western blotting using antibodies against phosphorylated or unphosphorylated FGFR1. Bands were quantified using densitometry. The *P* value was determined using one-way ANOVA with Tukey’s multiple comparisons test. **P < 0.01, ***P < 0.001 and ****P < 0.0001 for comparisons between the nonstimulated cells (0 minutes) and the hBD-3-stimulated cells without inhibitors. ^#^ *P* < 0.05 and ^####^ *P* < 0.0001 for comparisons between the hBD-3-stimulated cells in the presence or absence of inhibitor, *n* = 3.

Wound healing relies on angiogenic growth factors, and degradation of these factors or downregulated expression of their receptors impairs both angiogenesis and wound healing (3, 6). To verify whether hBD-3 mediated angiogenesis *via* angiogenic factor receptors, we pretreated fibroblasts with SSR 128129E (FGFR inhibitor), fruquintinib (VEGFR inhibitor) and imatinib mesylate (PDGFR inhibitor) and tested production of FGF, PDGF and VEGF after hBD-3 stimulation. Interestingly, among the above inhibitors tested, the FGFR inhibitor SSR 128129E exhibited the most significant inhibitory effect on PDGF and VEGF production but failed to inhibit FGF production (**Figure 6A**). Pretreating fibroblasts with various concentrations of fruquintinib and imatinib mesylate for 2 to 24 hours did not significantly reduce hBD-3-induced production of the tested angiogenic growth factors, suggesting that VEGFR and PDGFR were not involved in hBD-3-mediated production of angiogenic growth factors by fibroblasts (**Figures S2, S3**).

Next, we examined the effect of the FGFR pathway on cell migration and proliferation. Pretreatment of fibroblasts with SSR 128129E markedly delayed *in vitro* wound healing (**Figure 6B**) and suppressed fibroblast chemotaxis (**Figure 6C**) and cell proliferation (**Figure 6D**) induced by hBD-3. These results suggest that the FGFR pathway mediates the hBD-3-induced promotion of angiogenesis and wound healing. Indeed, we confirmed that hBD-3 induced FGFR activation. As shown in **Figure 6E**, stimulation of fibroblasts with hBD-3 showed that this peptide significantly induced the phosphorylation of FGFR1 threefold and fourfold following stimulation for 60 minutes and 120 minutes, respectively. This result suggests that hBD-3 upregulates the activity of the FGFR1 receptor mainly through phosphorylation.

### hBD-3 Induces Phosphorylation of JAK2 and STAT3

**Figure 7:**
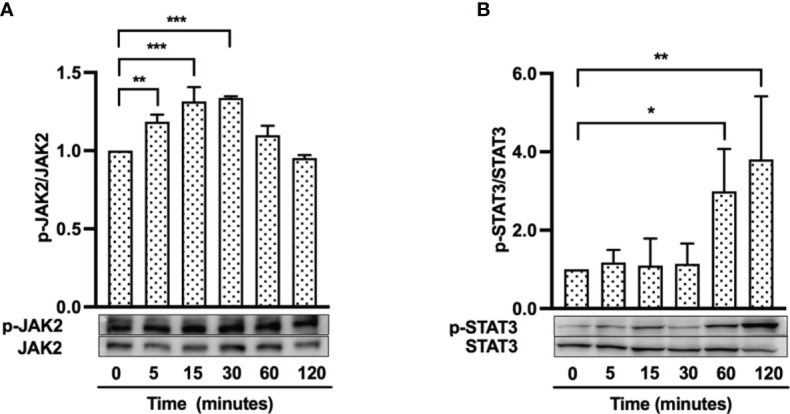
hBD-3 enhances phosphorylation of JAK2 and STAT3. Fibroblasts were stimulated with 20 µg/ml hBD-3 for 5 minutes to 120 minutes and subjected to Western blotting using antibodies against phosphorylated or unphosphorylated JAK2 **(A)** and STAT3 **(B)**. Bands were quantified using densitometry. The *P* value was calculated using one-way ANOVA with Tukey’s multiple comparisons test. *P < 0.05, **P < 0.01, and ***P < 0.001 for comparisons between the nonstimulated (0 minutes) and hBD-3-stimulated cells. *n* = 3.

Because the FGFR/JAK/STAT pathway is known to contribute to cell proliferation, angiogenesis and migration during wound healing (38), we hypothesized that hBD-3 might activate the JAK and STAT pathways, in addition to the FGFR pathway. Using Western blotting, we observed that hBD-3 induced phosphorylation of both JAK2 and STAT3. Following stimulation with hBD-3, JAK2 underwent rapid phosphorylation, which was already detectable after 5 minutes of stimulation. This phosphorylation peaked at 15 minutes before decreasing to basal levels within 2 hours (**Figure 7A**). In contrast to JAK2, hBD-3-induced STAT3 phosphorylation was slightly detected at 60 minutes and strongly elevated at 120 minutes (**Figure 7B**). These results indicate that JAK2 and STAT3 are part of the molecular pathways involved in hBD-3-mediated activation of fibroblasts. We observed that hBD-3 did not affect the phosphorylation of JAK1, JAK3, or STAT1 (**Figure S4**).

### Both JAK2 and STAT3 Are Necessary for the hBD-3-Mediated Production of Angiogenic Growth Factors, Migration, and Proliferation in Fibroblasts

**Figure 8:**
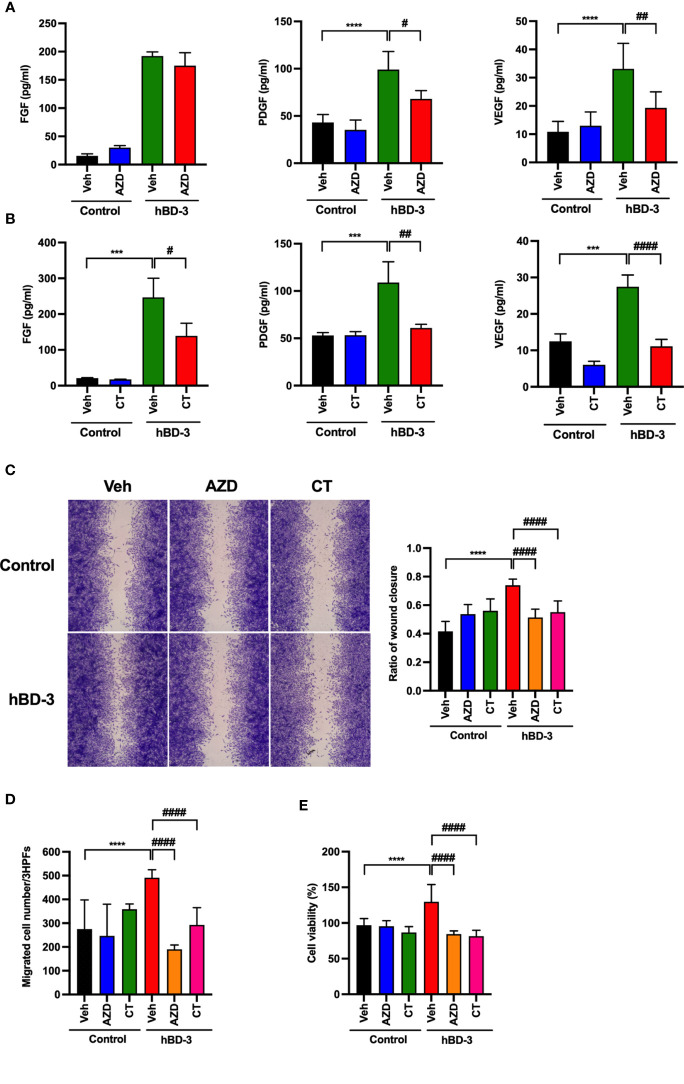
Both JAK2 and STAT3 are necessary for the hBD-3-mediated production of angiogenic growth factors, migration and proliferation of fibroblasts. Fibroblasts were pretreated with **(A)** 10 µM AZD1480 (AZD) or **(B)** 8 µM cryptotanshinone (CT) or 0.1% DMSO (vehicle) for 2 hours and then exposed to hBD-3. The levels of VEGF, PDGF and FGF in the culture supernatants from pretreated cells stimulated for 48 hours with 20 µg/ml hBD-3 were measured by appropriate ELISAs. **(C)** An *in vitro* wound scratch assay in pretreated fibroblasts stimulated with solvent (control) or 0.25 µg/ml hBD-3 for 24 hours was performed. Left panels show representative images, while the right panel represents the average of wound areas analyzed using ImageJ software. **(D)** Pretreated fibroblasts were also added to the upper wells of the microchamber and allowed to migrate for 6 hours towards solvent (control) or 0.5 µg/ml hBD-3. Migrated cells were counted in 3 random high-power fields (HPFs) under a light microscope. **(E)** Pretreated fibroblasts were stimulated with 1.0 µg/ml hBD-3 for 72 hours, and cell proliferation was assessed using the CCK-8 kit. The *P* value was determined using one-way ANOVA with Tukey’s multiple comparisons test. ***P < 0.001 and ****P < 0.0001 for comparisons between the nonstimulated cells (control) and the hBD-3-stimulated cells without inhibitors. ^#^ *P* < 0.05, ^##^ *P* < 0.01 and ^###^ *P* < 0.001 for comparisons between the hBD-3-stimulated cells in the presence or absence of inhibitor, *n* = 3.

To determine whether the JAK2/STAT3 pathways are indeed required for hBD-3-induced angiogenic growth factor secretion, migration and proliferation of fibroblasts, cells were pretreated with AZD1480 (JAK2 inhibitor) or cryptotanshinone (STAT3 inhibitor) for 2 hours before stimulation with hBD-3 for 24 hours. As shown in **Figure 8A**, the presence of AZD1480 markedly reduced the hBD-3-induced production of PDGF and VEGF by approximately 60% and 50%, respectively. However, the presence of AZD1480 did not affect hBD-3-mediated FGF production. AZD1480 had no significant effect on the spontaneous production of FGF, PDGF and VEGF. Similarly, we observed that pretreatment of fibroblasts with the STAT3 inhibitor cryptotanshinone suppressed the hBD-3-mediated production of both VEGF and PDGF to basal levels and reduced the production of FGF by approximately 50% (**Figure 8B**).

We next evaluated the inhibitory effects of JAK2 and STAT3 inhibitors on fibroblast migration and proliferation. Pretreatment of fibroblasts with either AZD1480 or cryptotanshinone noticeably suppressed the *in vitro* wound healing promoted by hBD-3 by approximately 40% or 50%, respectively (**Figure 8C**). Moreover, both AZD1480 and cryptotanshinone reduced fibroblast chemotaxis and proliferation induced by hBD-3 at the basal levels (**Figures 8D, E**). Collectively, these data demonstrated that hBD-3 promotes angiogenesis and wound healing through the FGFR1/JAK2/STAT3 pathways. Indeed, we confirmed that the FGFR inhibitor also inhibited hBD-3-induced phosphorylation of JAK2 and STAT3, suggesting that the JAK2/STAT3 pathways function downstream of FGFR1 (**Figure S5**).

## Discussion

**Figure 9:**
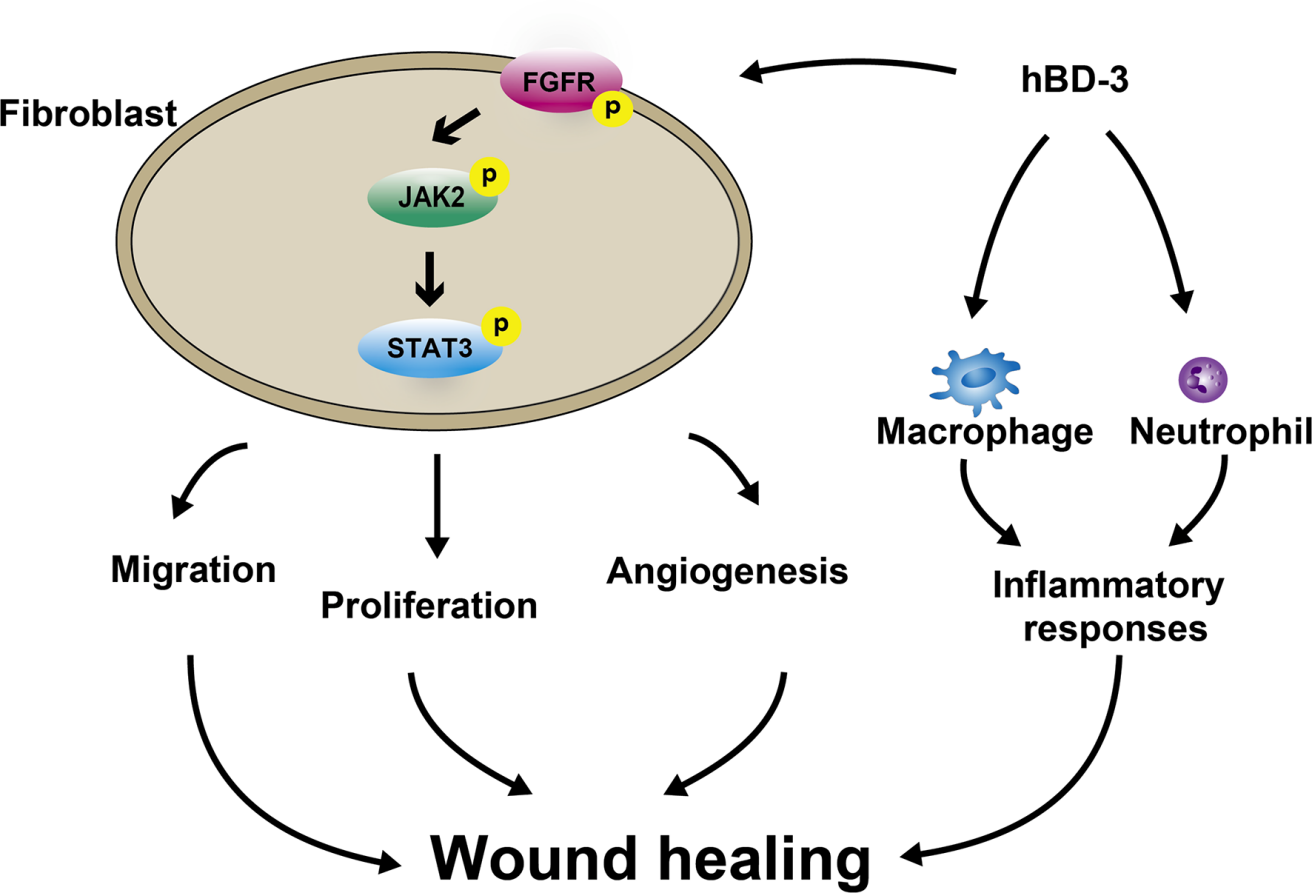
Schematic illustrating how hBD-3 is involved in wound healing. hBD-3 induces accumulation of neutrophils and macrophages in the early phase of wound healing and reduction of these phagocytes in the latter phase, leading to regulation of inflammatory responses during the wound healing process. hBD-3 also stimulates fibroblasts to migrate, proliferate and promote angiogenesis *via* activation FGFR/JAK2/STAT3 pathways.

Previous studies showed that the skin-derived antimicrobial peptide hBD-3 chemoattracts macrophages, mast cells and keratinocytes that contribute to the wound healing process (19, 39); however, the effects of hBD-3 on fibroblasts have not been reported thus far. In this investigation, following hBD-3 treatment, the wounds created in C57BL/6 mice healed faster, showed accumulation of inflammatory cells in the early stage of wound healing, and increased the number of fibroblasts and newly formed vessels. Additionally, *in vitro* studies demonstrated that hBD-3 enhanced the production of various angiogenic growth factors and induced the migration and proliferation of human fibroblasts through the FGFR1/JAK2/STAT3 pathways (**Figure 9**). These observations suggest that hBD-3 might accelerate skin wound healing *via* activation of fibroblasts.

Although we used mice as an animal model to investigate the therapeutic effects of hBD-3 on wound healing, wound healing in mice is basically different from that in humans because it mainly occurs *via* contraction (40). To avoid and prevent contraction, we used ring-shaped silicone splints, thus allowing the repair process to be dependent on epithelialization, cellular proliferation and angiogenesis, a process similar to that in humans (41). In the inflammatory phase of wound healing, innate immune cells such as macrophages and neutrophils accumulate at the wound site to attack invading pathogens and remove dead tissues (42, 43). In this phase, the number of infiltrating macrophages reaches a peak 3 days post-injury and persists until day 7, whereas the number of neutrophils peaks 1 day post-wounding before declining gradually (44). Moreover, macrophages are divided into M1 and M2 phenotypes. M1 phenotype appears in the very early stage of inflammation with pro-inflammatory properties, whereas M2 phenotype is predominant in the latter stage of inflammation with anti-inflammatory effects (30, 31). Additionally, M2 macrophages are involved in wound healing by promoting angiogenesis *via* the production of VEGF and remodeling (45). Transition of macrophages from M1 to M2 phenotype occurs during acute wound healing process, and failure of this transition leads to the persistence of pro-inflammatory macrophages at the wound site, resulting in chronic wound (30, 31, 46). In our study, we observed that in hBD-3-treated wounds, the number of both neutrophils and macrophages, mostly M2 but not M1 phenotype, at the wound site was higher in the early stage of wound healing (day 2 post-injury) and declined on day 4, whereas the number of these cells increased at day 4 in the control groups. This observation suggested that hBD-3 initiates the inflammatory phase and contributes to inflammatory resolution, leading to acceleration of the wound healing process. Chronic wounds, including diabetic foot ulcers are often linked to prolonged inflammation, which may be caused by various factors such as dysfunction of neutrophils and macrophages, the failure of macrophages to switch from M1 to M2 phenotype, reduction of anti-inflammation cytokines and overproduction of reactive oxygen species (47–50). The ability of hBDs to induce the migration and activation of neutrophils and macrophages (51–53) might be one of the mechanisms by which it accelerates the infiltration of phagocytes, leading to wound healing.

Among the cells involved in the wound healing process, fibroblasts play a crucial role from the late inflammatory phase until the remodeling phase through migration, proliferation, and secretion of growth factors, cytokines, collagens and other ECM components (54). In the current study, hBD-3-treated wounds displayed an increased number of fibroblasts compared with those of the control mice and showed increased expression of angiogenic growth factors such as FGF, PDGF and VEGF. These growth factors were also significantly secreted in human fibroblasts following hBD-3 stimulation. Notably, VEGF is the most effective angiogenic factor that accelerates neovascularization, angiogenesis and epithelialization (6, 55). FGF is a key regulator of angiogenesis and contributes to re-epithelialization, whereas PDGF promotes cell migration and proliferation (6, 55, 56). hBD-3-stimulated fibroblasts also showed improved migration and proliferation, the two most essential steps responsible for wound closure (57). In addition to growth factors, fibroblasts generate various MMPs. Among the MMPs, MMP-2 and MMP-9 cleave ECM, cytokines, growth factors, and cytokine/growth factor receptors, which control and coordinate signaling pathways in cell proliferation, migration, inflammation, and angiogenesis (37, 58). We found that hBD-3 increased the expression of MMP-2 but not MMP-9. MMP-2 has been shown to be indispensable for angiogenesis and prolonged matrix remodeling, while MMP-9 is involved in keratinocyte migration and granulation tissue remodeling (2).

Wound healing relies on interactions of various growth factors and cytokines that regulate cellular responses. Angiogenic growth factors promote cell proliferation, migration, and angiogenesis by binding to their corresponding receptors and activating multiple downstream signaling pathways (59). Previous studies have shown that the levels of angiogenic growth factors such as FGF, PDGF and VEGF and their respective receptors are downregulated in nonhealing chronic wounds (11–13). Therefore, it is presumed that activation of angiogenic factor receptors along with their downstream pathways may represent a promising novel approach for the treatment of nonhealing chronic wounds. Indeed, some angiogenic growth factors, including FGF, PDGF and VEGF, have been used in clinical trials to promote tissue repair (60–62). Based on this evidence, we investigated the role of hBD-3 in promoting angiogenesis, cell proliferation, and migration of fibroblasts through activation of growth factor receptors such as FGFR, PDGFR and VEGFR. Among the specific inhibitors of the above receptors tested, only the FGFR inhibitor suppressed hBD-3-mediated fibroblast activation, suggesting an important role of FGFR in hBD-3-mediated fibroblast activation. Following binding to FGF, FGFR undergoes autophosphorylation to initiate angiogenesis and tissue remodeling through downstream signaling pathways (3, 4, 6). Our study revealed that hBD-3 significantly increased FGFR1 phosphorylation and that blocking this receptor with a specific inhibitor markedly suppressed the secretion of angiogenic growth factors, cell migration and proliferation in human fibroblasts.

The JAK/STAT pathway constitutes a major signaling mechanism for various growth factors and cytokines in mammals (63) and plays roles in processes such as cellular proliferation, differentiation, immune regulation, inflammatory response and angiogenesis (64, 65). Among the JAK members, JAK1, 2, and 3 are broadly detected in various tissues and cells, whereas TYK3 is only found in the bone marrow and lymph system (63). After their activation, JAKs induce the phosphorylation of STAT molecules. In this study, hBD-3 induced the phosphorylation of JAK2 but not that of JAK1 and JAK3 and enhanced the phosphorylation of STAT3 but not that of STAT1. Among STAT members, STAT3 has been shown to be important in wound healing through its ability to promote angiogenesis under both physiological and pathological conditions in addition to cell survival, proliferation, and differentiation (66). In this study, the upstream cascade of the JAK2/STAT3 pathway involved FGFR1, as evidenced by the inhibitory effect of an FGFR inhibitor on JAK2 and STAT3 phosphorylation by hBD-3. This inhibitor also suppressed fibroblast migration, proliferation, and secretion of angiogenic growth factors. In a previous study, an association between FGFR and STAT3 activation was demonstrated and was dependent on JAK2 (67).

Following tissue injury, the wound site becomes hypoxic due to the disruption of blood vessels. Hypoxia is a crucial moderator of normal wound healing as it influences every wound healing stage, from fibroblast proliferation to tissue growth and remodeling (68). Therefore, a failure to respond to hypoxic stimuli due to the deficiency of hypoxia-inducible factor (HIF)-1α may lead to the formation of chronic wounds (68). Although there is no direct evidence demonstrating hBD-3-induced activation HIF-1α to accelerate wound healing, the findings that major antimicrobial peptides such as defensins and cathelicidins are downregulated in HIF-1α-deficient mice suggest a correlation between antimicrobial peptides and HIF-1α activation (69). Furthermore, the fact that hBD-3 activates the nuclear factor-kappa B (NF-κB) pathway (70), which interacts with HIF-1α pathway (71) further suggests possible contribution of hBD-3 to HIF-1α activation.

In conclusion, the findings of our study suggest the therapeutic effect of the skin-derived antimicrobial peptide hBD-3 in wounds through its ability to promote wound healing, angiogenesis, cell proliferation and migration in fibroblasts. The effects of hBD-3 on fibroblasts were mediated *via* the FGFR1/JAK2/STAT3 signaling pathways.

## Data Availability Statement

The raw data supporting the conclusions of this article will be made available by the authors, without undue reservation.

## Ethics Statement

The animal study was reviewed and approved by Institutional Review Committee of Juntendo University, Juntendo University Graduate School of Medicine.

## Author Contributions

MT and YU performed experiments and data analysis, and wrote the manuscript. HY, JT-P, GP, LN, and RI assisted with data acquisition and analyses. KO, HO, SI, and FN contributed to conceptualization, methodology, and writing the manuscript. All authors contributed to the article and approved the submitted version.

## Funding

Parts of the work presented here were supported by a Grant-in-Aid for Scientific Research from the Ministry of Education, Culture, Sports, Science and Technology, Japan (Grant number: 21K08309 to FN) and by the Atopy (Allergy) Research Center, Juntendo University, Tokyo, Japan.

## Conflict of Interest

The authors declare that the research was conducted in the absence of any commercial or financial relationships that could be construed as a potential conflict of interest.

## Publisher’s Note

All claims expressed in this article are solely those of the authors and do not necessarily represent those of their affiliated organizations, or those of the publisher, the editors and the reviewers. Any product that may be evaluated in this article, or claim that may be made by its manufacturer, is not guaranteed or endorsed by the publisher.
