# Supplementary material for: The Antimicrobial Peptide Human β-Defensin-3 Accelerates Wound Healing by Promoting Angiogenesis, Cell Migration, and Proliferation Through the FGFR/JAK2/STAT3 Signaling Pathway
Source: Front Immunol. 2021 Sep 14;12:712781. doi: 10.3389/fimmu.2021.712781 (PMC8476922; doi:10.3389/fimmu.2021.712781)
Supplement: Supplementary file 1 [file DataSheet_1.pdf]

frontiers in Immunology (Original Research)

The antimicrobial peptide human β-defensin-3 accelerates wound healing by promoting angiogenesis, cell migration and proliferation through the FGFR/JAK2/STAT3 signaling pathway

Miho Takahashi[1,2], Yoshie Umehara[1], Hainan Yue[1,2], Juan Valentin Trujillo-Paez[1], Ge Peng[1,2], Hai Le Thanh Nguyen[1,2], Risa Ikutama[1,2], Ko Okumura[1], Hideoki Ogawa[1,2], Shigaku Ikeda[1,2], François Niyonsaba[1,3*]

[1]Atopy (Allergy) Research Center, Juntendo University Graduate School of Medicine, Tokyo, Japan

[2]Department of Dermatology and Allergology, Juntendo University Graduate School of Medicine, Tokyo, Japan

[3]Faculty of International Liberal Arts, Juntendo University, Tokyo, Japan

*** Correspondence:**

François Niyonsaba, Atopy (Allergy) Research Center and Faculty of International Liberal Arts, Juntendo University, 2-1-1 Hongo, Bunkyo-ku, Tokyo 113-8421, Japan

Email address: francois@juntendo.ac.jp

Telephone number: +81-3-5802-1591; Fax number: +81-3-3813-5512

Keywords: angiogenesis, human β-defensin, fibroblast, migration, proliferation, wound healing

Running title: Human β-defensin-3 promotes wound healing

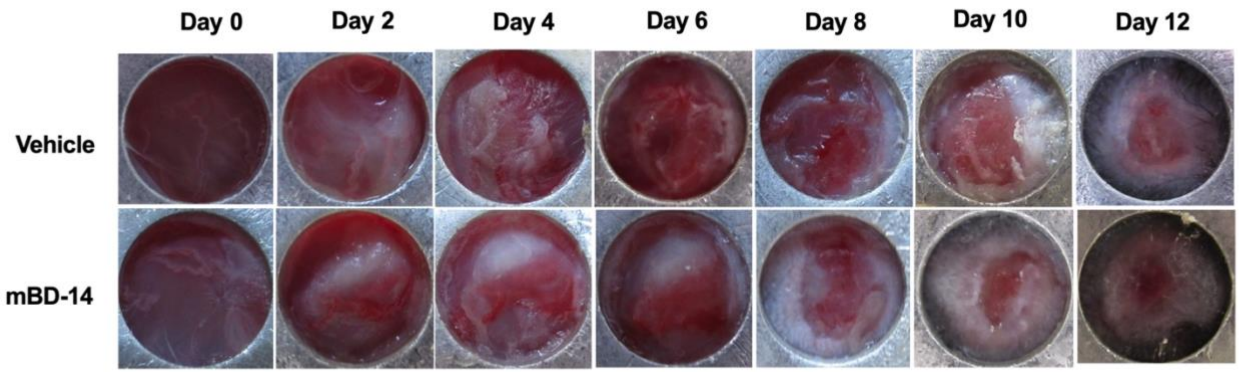

Figure S1. mBD-14 accelerates wound closure in vivo

Dorsal full-thickness skin wounds were created on mice and then topically treated with 0.01% acetic acid (vehicle) and 200 μg/ml mBD-14. Representative images of skin wounds from day 0 to day 12.

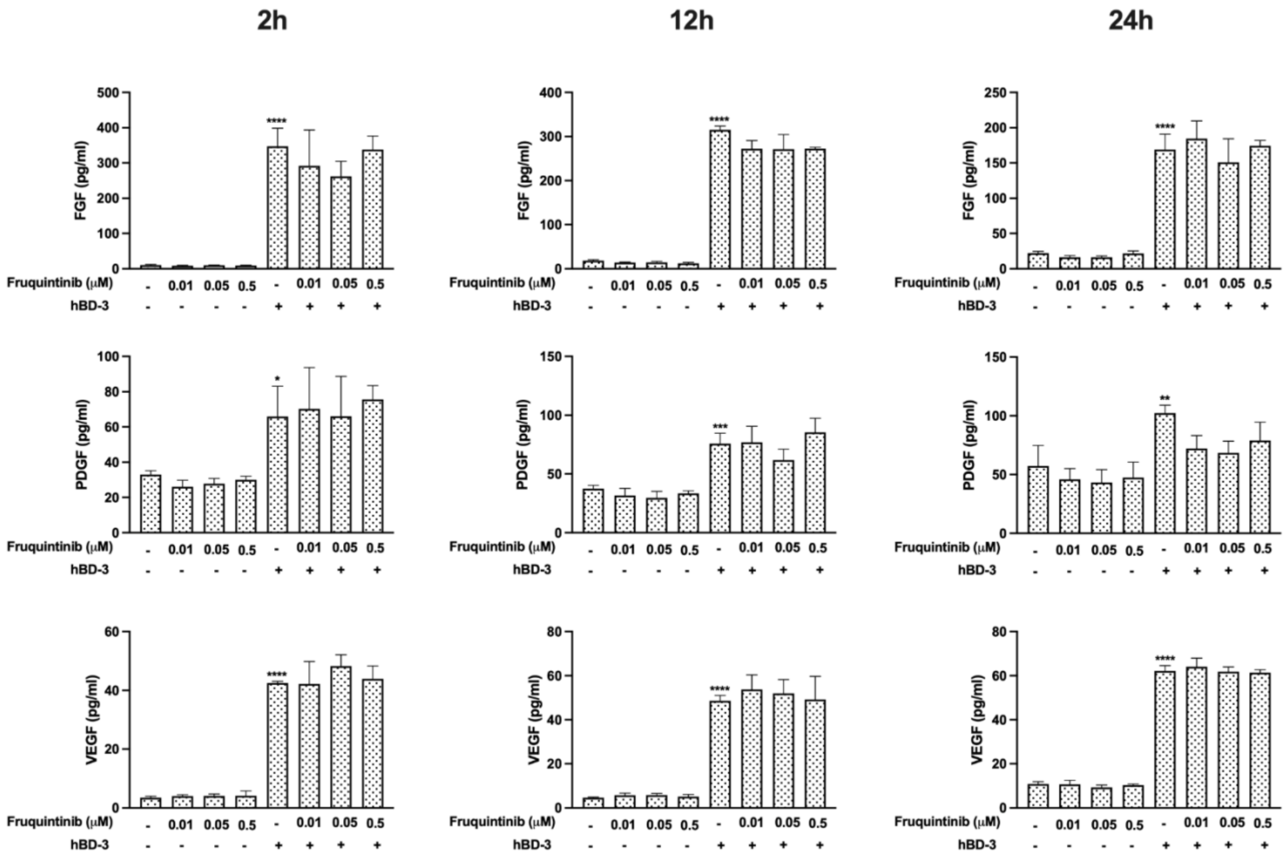

Figure S2. VEGFR is not involved in hBD-3-mediated production of angiogenic growth factors

Fibroblasts were pretreated with 0.1% DMSO (vehicle) or 0.01 to 0.5 μM fruquintinib (VEGFR inhibitor) for 2 to 24 hours and then exposed to 20 μg/ml hBD-3. The amounts of VEGF (upper panels), PDGF (middle panels) and FGF (lower panels) in culture supernatants were measured by appropriate ELISAs. The *P* value was determined using one-way ANOVA with Tukey's multiple comparisons test. * $P < 0.05$, ** $P < 0.01$, *** $P < 0.001$, and **** $P < 0.0001$ for comparisons between the nonstimulated cells and the hBD-3-stimulated cells without inhibitors. NS (not significant) for comparisons between the hBD-3-stimulated cells in the presence or absence of inhibitor, $n = 3$.

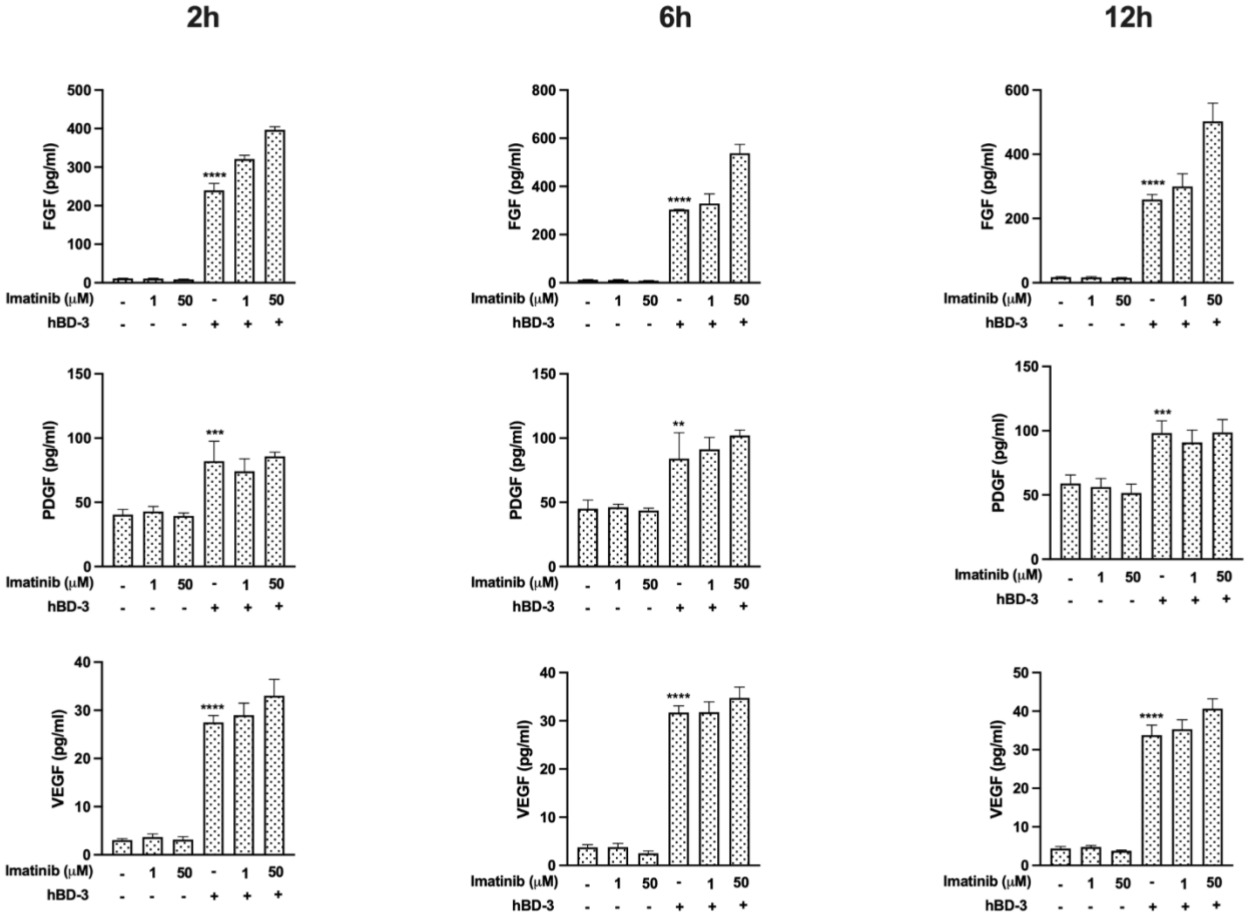

Figure S3*. *PDGFR is not required for hBD-3-mediated production of angiogenic growth factors

Fibroblasts were pretreated with 0.1% DMSO (vehicle) or 1 to 50 μM imatinib (PDGFR inhibitor) for 2 to 12 hours and then exposed to 20 μg/ml hBD-3. The amounts of VEGF (upper panels), PDGF (middle panels) and FGF (lower panels) in culture supernatants were measured by appropriate ELISAs. The *P* value was determined using one-way ANOVA with Tukey's multiple comparisons test. ** $P < 0.01$, *** $P < 0.001$, and **** $P < 0.0001$ for comparisons between the nonstimulated cells and the hBD-3-stimulated cells without inhibitors. NS (not significant) for comparisons between the hBD-3-stimulated cells in the presence or absence of inhibitor, $n = 3$.

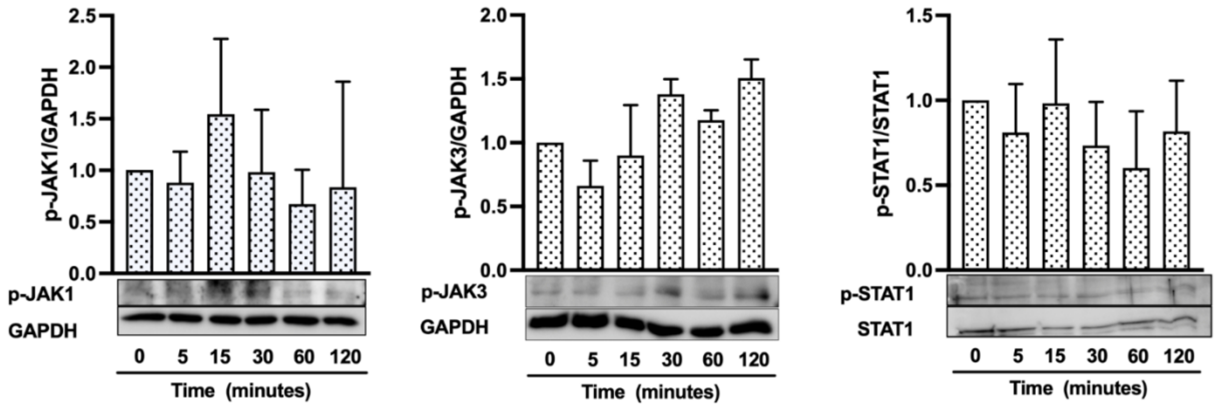

Figure S4. hBD-3 does not affect the phosphorylation of JAK1, JAK3 and STAT1

Fibroblasts were stimulated with 20 μg/ml hBD-3 for 5 minutes to 120 minutes and then subjected to Western blotting using antibodies against phosphorylated or unphosphorylated JAK1, JAK3 and STAT1. 0 minutes: nonstimulated cells. Bands were quantified using densitometry.

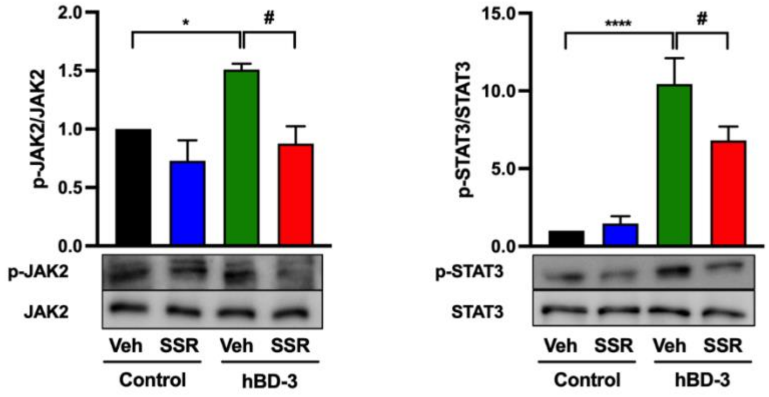

Figure S5. FGFR inhibitor suppresses hBD-3-induced phosphorylation of JAK2 and STAT3

Fibroblasts were pretreated with 0.1% DMSO (vehicle) or 1 μM SSR 128129E (FGFR inhibitor, SSR) for 2 hours and then exposed to 20 μg/ml hBD-3 for 15 minutes (JAK2) and for 120 minutes (STAT3) and subjected to Western blotting using antibodies against phosphorylated or unphosphorylated JAK2 (A) and STAT3 (B). Bands were quantified using densitometry. *P* value was conducted using one-way ANOVA with Tukey's multiple comparisons test. * $P < 0.05$ and *** $P < 0.001$ for comparisons between the nonstimulated cells (Control) and the hBD-3-stimulated cells without inhibitors. # $P < 0.05$ for comparisons between hBD-3-stimulated cells in the presence or absence of inhibitor, $n = 3$.
